# Genetic counselors' response types to prenatal patient deferring or attributing religious/spiritual statements: An exploratory study of US genetic counselors

**DOI:** 10.1002/jgc4.1634

**Published:** 2022-09-21

**Authors:** Alina Sitaula, Patricia McCarthy Veach, Ian M. MacFarlane, Whiwon Lee, Krista Redlinger‐Grosse

**Affiliations:** ^1^ Invision Sally Jobe Greenwood Village Colorado USA; ^2^ University of Minnesota Minneapolis Minnesota USA; ^3^ Department of Genetics, Cell Biology, and Development, Institute of Human Genetics University of Minnesota Minneapolis Minnesota USA; ^4^ Institute of Health Policy, Management and Evaluation University of Toronto Toronto Ontario Canada

**Keywords:** cultural competence, genetic counseling, psychosocial, religion, spirituality

## Abstract

Research shows religiosity and spirituality (R/S) influence genetic counseling patients' and families' risk perception, decision‐making, and coping. No published studies have examined how genetic counselors respond to patient‐initiated R/S statements. This exploratory study examined genetic counselors' response types and reasons for their responses to two prenatal patient's R/S statements. Genetic counselors (*n* = 225) recruited through a National Society of Genetic Counselors eblast completed a survey containing two hypothetical scenarios regarding a prenatal patient's receipt of a trisomy 18 diagnosis. Scenarios were identical except for the last patient statement: “God makes everything possible…we leave things in his hands” (a deferring statement) or “I feel like God is punishing me for something I did” (an attributing statement). Imagining they were the counselor, participants wrote a response to each scenario and provided reasons for their response. Responses were analyzed using the Helping Skills Verbal Response System. MANOVA and chi‐square tests, examining differences in response type based on patient statement (deferring or attributing), participant comfort with R/S, and years of experience, yielded a significant multivariate effect for scenario (*p* < 0.001). Responses to the deferring statement scenario contained a greater proportion of content statements (*p* < 0.001), closed questions (*p* < 0.001), and information‐giving (*p* < 0.001). Responses to the attributing statement scenario contained a greater proportion of open questions (*p* = 0.05), influencing statements (*p* < 0.001), and affective statements (*p* = 0.006). Neither comfort with R/S nor genetic counseling experience significantly affected response type. Thematic analysis of reasons for responses yielded nine themes. Most prevalent were exploration (of the patient's statement), validation, correction (of patient's beliefs), and reassurance. The findings reflect stylistic differences in how and why genetic counselors respond to patients.

**What is known about this topic**

Studies have explored the use of religion/spirituality (R/S) by patients, but none have directly examined genetic counselors' response types to R/S statements.

**What this paper adds to the topic**

Findings illustrate the wide variety of genetic counselor response types to patient deferring and attributing patient statements and suggest variables warranting further research.

## 1 INTRODUCTION

Religiosity and spirituality (R/S) are key aspects of life for a substantial portion of the U.S. population. An estimated 50% report religion as personally very important, and a 2016 Gallup poll indicates 89% of Americans believe in God or a universal spirit (“Religion Gallup Historical Trends,” 2017). R/S seem to be particularly important to people when dealing with stressful life events (Bjorck & Thurman, 2007). Healthcare studies have found religion and spirituality play a key role for some patients with respect to decision‐making, coping, emotional well‐being, recovery, and treatment compliance (Ehman et al., 1999; Grossoehme et al., 2010; Hodge, 2011; Medved Kendrick, 2017; Schwartz et al., 2000). Puchalski et al. (2014) speak to the importance of incorporating spirituality in healthcare to support and improve health outcomes and honor “the dignity of each person” (p. 642). Therefore, patient‐centered approaches warrant the incorporation of religious and spiritual beliefs into some patients' care plans.

The relevance of R/S may intensify in genetic counseling situations, as patients often encounter uncertain genetic risk and information and/or face difficult decisions (e.g., Quillin et al., 2006; White, 2009). While patients with religious and spiritual inclinations may draw upon their faith in the face of genetic risks or diagnoses, little is known about how genetic counselors address patients' beliefs during a counseling session. The purpose of this study is to explore genetic counselors' responses to two different ways a hypothetical prenatal patient brings up her R/S in relation to a genetic condition.

### 1.1 Definitions of religiosity and spirituality

Religiosity is often defined as adherence to a particular set of beliefs, participation in prayers or worship, upholding religious traditions, and/or affiliation with a specific religious community (Knapp et al., 2010). Historically, spirituality was considered a process that unfolds within a religious context (Steinhauser et al., 2017). While contemporary definitions of spirituality vary, they generally include an emphasis on a sense of purpose derived from a relationship with a higher power and reliance on personal moral and ethical standards (Knapp et al., 2010; Puchalski et al., 2009). R/S overlap in that religious individuals are often spiritual, and spirituality is commonly, though not always, expressed through religious practices and traditions (Knapp et al., 2010; Puchalski et al., 2009; Steinhauser et al., 2017).

### 1.2 Effects of R/S on patients and families at genetic risk

Several studies have examined the influence of R/S on patients' risk perception, decision‐making, and coping in relation to their own or their family members' genetic risks, diagnoses, and testing (e.g., Cotton et al., 2012; Ehman et al., 1999; Quillin et al., 2006; Sagaser et al., 2016; Seth et al., 2011; Schwartz et al., 2000). For instance, patients in prenatal and preconception genetic counseling have reported using their R/S to make decisions in their pregnancy and to deal with stressors (Sagaser et al., 2016). Seth et al. (2011) examined the role of R/S in decision‐making for amniocentesis in pregnancy. They found that although most of the participants' decisions about amniocentesis were heavily influenced by procedural‐related risks for complications, they did rely on their faith and beliefs for comfort after making a decision. Themes in responses from all participants included “faith in the existence of God” and using their hope and faith as “pillars of support” (p. 665). Women with different obstetric risks have been found to use spiritual coping to deal with the various stresses of their pregnancies (Breen et al., 2006; Hamilton & Lobel, 2008).

### 1.3 Patient perceptions of R/S exploration by healthcare providers

Research indicates that patients with and without religious beliefs generally welcome physicians' inquiry about their R/S beliefs (Daalemann & Nease, 1994; Ehman et al., 1999). Two genetic counseling studies demonstrated that patients generally are comfortable sharing their beliefs in sessions and would welcome the genetic counselor incorporating R/S into the discussion (Sagaser et al., 2016; Thompson et al., 2016). Sagaser et al. (2016) also found that genetic counselors were significantly more likely to identify R/S as important to a patient when the patient used religious/spiritual language (faith, belief, blessing, sin, trust, God's will, God's hands, etc.). In contrast, in the absence of such language, genetic counselors were usually uncertain about the importance of R/S to the patient.

Although research suggests some patients are comfortable with R/S discussions in genetic counseling session, one study found only about 60% genetic counselors reported performing a spiritual assessment in the previous year, and only about 8% of those counselors conducted the assessments in greater than half of their sessions (Reis et al., 2007). The most commonly reported reasons for such assessment were because the patient initiated the topic, and the session involved termination and end‐of‐life issues. Frequently reported reasons for lack of assessment were low perceived relevance of spiritual assessment to genetic counseling and counselor discomfort.

### 1.4 Purpose of the study

Prior research supports the importance of R/S to many individuals in the U.S. and R/S and coping may be particularly prevalent in times of stress. Furthermore, some research suggests patients are comfortable being asked about and/or sharing their R/S beliefs with healthcare providers, including genetic counselors. While studies to date have explored the use of R/S by patients in the context of genetic risks, diagnoses, and testing, as well as patients' and genetic counselors' perceptions of R/S exploration in genetic counseling sessions, no research has directly examined genetic counselors' responses to patient‐initiated R/S discussions. The present survey study explored the types of responses genetic counselors make to prenatal patient‐initiated R/S statements. There are two major research questions and three sub‐questions:

1. How do genetic counselors respond to prenatal patient‐initiated statements about her R/S beliefs in relation to a genetic condition?
  - Do response types differ as a function of the content of the patient's R/S statements?
  - Do response types differ due to general comfort level discussing R/S issues?
  - Do response types differ due to years of experience?
2. What reasons do the genetic counselors give for their response types to the patient statements?

This exploratory study begins to address a gap in the literature by identifying genetic counselor response types to a hypothetical prenatal patient bringing up her R/S in relation to a genetic condition.

## 2 METHOD

### 2.1 Participants

Upon receipt of approval from the University of Minnesota Institutional Review Board (STUDY00004207), genetic counselors were recruited through a National Society of Genetic Counselors (NSGC) eblast (~*n* = 3876). An invitation described the study as an investigation of how genetic counselors respond to patients' religious or spiritual statements with the goal of describing current practices and informing recommendations for current and future professionals. The invitation contained a link to an anonymous online survey. The initial study invitation was sent in October 2018, and a reminder email was sent 5 weeks later.

### 2.2 Instrumentation and procedures

We developed an online survey (See supplemental appendix) comprising three sections. The first section reviewed the purpose of the study, consent information, and survey expectations. The second section contained two hypothetical prenatal genetic counseling scenarios in which Non‐Invasive Prenatal Test and level 2 ultrasound findings for the patient are consistent with trisomy 18. The genetic counselor is discussing with patient whether she wishes to pursue amniocentesis.

The scenarios and genetic counselor‐patient dialogue were identical with the exception of the final patient statement which was either a deferring R/S statement or an attributing R/S statement. We created these statements based on our clinical experience that they are prevalent among prenatal patients who express R/S beliefs, as well as studies that have found a belief in God's will are a common theme among Hispanic patients, with R/S beliefs that accept or decline prenatal testing options ( Browner et al., 1999). God's will has been cited as referencing both acceptance of a health outcome, as well as a belief that God will assist them regardless of the outcome and/or God's power in a child's wellbeing (Rehm, 1999). As stated by Seth et al. (2011), “families not only believed in God's help, but felt that the outcome was also influenced by their own contributions to the child's health care needs” (p. 662). Thus, the scenarios and R/S statements in the present study are based on an amalgamation of anecdotal and empirical information. For clarity, we herein reference each patient statement/scenario as “deferring” or “attributing.” The deferring R/S statement indicates the patient's reliance on an external entity. The attributing R/S statement expresses the patient's perception that she is the possible cause of the genetic condition. Deferring “implies a voluntary yielding or submitting out of respect or reverence and affection toward another”; attributing is the interpretive process of making judgments about cause and includes “an inferring or conjecturing of cause” (Merriam‐Webster, n.d.). Both responses were standardized with respect to word length, 20 and 21 words, respectively.

*Deferring Statement*: “I don't know. (Pause)… God makes everything possible. Only God knows what can happen. We leave things in his hands.” (Deferring R/S statement)

*Attributing Statement*: “I don't understand why this is happening to me. (Pause)… I feel like God is punishing me for something I did.” (Attributing R/S statement)?

Participants were asked to imagine themselves as the patient's genetic counselor and to compose a response to the R/S statement in each scenario as if they were speaking directly to her. After responding to the patient, they were asked to explain the rationale for their response. Presentation of the two scenarios was randomized and balanced to control for potential order effects.

The third section of the survey contained demographic questions (e.g., age, gender, ethnicity, genetic counseling experience, specialty area, whether they see patients, and if so, average number seen per week, and average hours worked per week). In addition to basic demographic information, participants were asked how often patients do/did bring up R/S issues (Scale: 1 = *Rarely/Never*, 2 = *Sometimes*, 3 = *Often*, 4 = *Very Often*) and their comfort level with R/S in sessions (Scale: 1 = *Little or not at all comfortable*, 2 = *Somewhat comfortable*, 3 = *Comfortable*, 4 = *Very Comfortable)*.

### 2.3 Data analyses

#### 2.3.1 Quantitative analyses

Descriptive statistics were calculated for responses to the demographic questions. Participants' responses in each scenario were analyzed using the Helping Skills Verbal Response System (HSVRS; Danish et al., 1980; Dotson, 1984). The HSVRS assesses the types of helping responses (rather than response quality) individuals use in one‐to‐one interactions. The HSVRS has demonstrated reliability and validity in studies of the helping responses of diverse groups, including paraprofessional helpers and untrained individuals (McCarthy et al., 1979); crisis interveners, psychotherapists, and nursing students (McCarthy & Knapp, 1984; Ryden et al., 1991); informal helpers (D'Augelli & Vallance, 2006); clinical supervisors (McCarthy et al., 1994); job recruiters (DeBell et al., 1998); and peers who reply to messages posted on grief websites (Swartwood et al., 2011).

P.MV. is a criterion‐trained rater in the HSVRS system, and she analyzed the participants' responses to each scenario. First, she divided each response into thought units (independent clauses that comprise a subject and a verb). Next, she classified each thought unit into one of nine types of responses, described in Table 1.

**TABLE 1:** Helping skills verbal response system (HSVRS) response types and examples of participant statements by scenario

Response type	Definition	Deferring scenario example (participant number)	Attributing scenario example (participant number)
Content	Reflect patient statements	It sounds like your faith is really important to you and that you would prefer to wait and see how things progress (2).	This was not something you had expected to have to deal with. (55)
Affect	Reflect feelings patient has not directly labeled	It sounds like your faith is bringing you a sense of comfort and peace at this time (172).	You are overwhelmed because you do not know why this has happened to you. (22)
Open Question	Require an extended answer	Could you tell me more about what you mean by “God makes everything possible?” (1)	What is it that you think God is punishing you for? (2)
Closed Question	Require 1‐ or 2‐word answer	Do you feel that believing to leave everything in God's hands helps you during the pregnancy? (41)	Do you feel like God punishes people in this way? (14)
Influence	Attempt to alter patient views; provide GC's opinion; encouragement	Doing the amnio allows for more information so that you have all the facts to make decisions. (119)	I want to let you know that there is nothing that you did that would have caused these test results. (1)
Advice	Suggest alternative behaviors	If that were the case, you may want to be sure to deliver in a hospital that can provide the appropriate level of care. (66)	The hospital also has a chaplain who would be happy to talk with you. (20)
Information	Provide facts or resources	Diagnostic testing, and knowing that trisomy 18 is present, would provide you with information on what to expect going forward in pregnancy. (50)	Our current understanding is that the risk of this happening increases as women get older. (7)
Self‐disclosure	Factual information based on GC's work experience; personal info about GC	Some of my patients have the amniocentesis, so that they have the answer and can prepare going forward – one way or another. (98)	I do not know very much about religion, but I feel like God loves people and would never want to punish them. (14)
Self‐Involving	Personal reactions to patient (usually GC feelings)	I am glad to hear that your spirituality is a source of strength, because I know this news can be difficult. (112)	I am so sorry that this news is so difficult. (4)

A one‐way multivariate analysis of variance (MANOVA) was conducted to check for potential order effects for any of the dependent variables. A 2x2 mixed MANOVA examined differences in total number of thought units and in proportionate use of the nine response types as a function of genetic counseling scenario, controlling for order effects. Proportions were used to control for differences in participant verbosity. They were obtained by dividing the frequency of each type of response by the total number of thought units. A 2x2 mixed MANCOVA was conducted to see whether controlling for genetic counselor experience level or self‐reported comfort discussing R/S issues affected the proportionate use of the nine response types.

A.S. reviewed all participant responses to the hypothetical patient and noted whether they mentioned R/S language (e.g., God, faith, religion, chaplain) based on R/S language denoted by Sagaser et al. (2016). A chi‐square test examined whether there was a significant difference in the percentage of participants who mentioned R/S language in their response for the Deferring scenario versus the Attributing scenario. Chi‐square tests were also done to examine whether mentioning R/S in either scenario was related to self‐reported comfort level discussing R/S issues or level of experience.

Given the exploratory nature of this study, it is important to balance protections for Type I errors (false positives) and Type II errors (false negatives), as falsely declaring no effect could be just as damaging to future research efforts as falsely declaring an effect. To this end, we assessed the two MANOVAs and the MANCOVA at α = 0.017 (Bonferroni correction for 3 tests) and the chi‐square analyses at α = 0.01 (Bonferroni correction for 5 tests) as a compromise between a strict Type I error correction strategy and maximizing power to identify promising areas for future research. Further Type I error corrections for post hoc tests (e.g., univariate ANOVAs for significant factors in the MANOVA or MANCOVA) were not conducted, as such measures are overly conservative given the significant preliminary test (e.g., Howell, 2010).

#### 2.3.2 Qualitative analysis

The participants' reasons for their responses to each scenario were analyzed using an inductive approach that looks for patterns, themes, and common categories without imposing a preexisting framework on the data (Silverman, 2000). A.S. and K.R.G. served as data analysts. Together, they coded the first 20 participant rationales from each scenario to extract themes for subsequent coding of all rationales. Then, A.S. coded the remaining rationales while continually reviewing thematic groupings. Following this step, K.R.G. audited her coding. They discussed any discrepancies to reach agreement.

## 3 RESULTS

### 3.1 Participant demographics

The recruitment process yielded 470 returned surveys (conservative response rate, assuming all members read the eblast = 12.1%). Of these, 225 participants responded to at least one scenario and were therefore included in the data analyses. Descriptive statistics for all study variables are shown in Tables 2 and 3. Participants were primarily female (90.7%) and White (87.6%), which is consistent with data from the 2018 NSGC Professional Status Survey (PSS; 95% female and 92% White; NSGC, 2018a). The 2018 PSS was used because it reflects NSGC membership at the time of data collection. Participants ranged in age from 24 to 72 years (*M* = 35.3, *SD* = 11.37) and had a mean of 7.58 years of experience (*SD* = 8.59). Work settings and practice specialties varied. The most prevalent work setting was a university medical center (33.3), and the most prevalent practice specialty was prenatal (34.7%). The most prevalent work setting on the 2018 PSS (NSGC, 2018b) was also university medical center (30%), though the most prevalent practice specialty was cancer (46%).

**TABLE 2:** Participant demographics (*N* = 225)

Variable	*n*	%
Gender		
Female	204	90.7
Male	9	4.0
Undisclosed	12	5.3
Ethnicity		
Caucasian or White	197	87.6
Asian	5	2.2
Biracial	4	1.8
Hispanic/Chicano/Latino(a)	2	0.9
Black/African‐American	1	0.4
Native Hawaiian/Pacific Islander	1	0.4
Other	1	0.4
Undisclosed	14	6.2
Comfort Level Discussing R/S with Patients		
Little or not at all comfortable	19	8.4
Somewhat comfortable	82	36.4
Comfortable	74	32.9
Very comfortable	36	16.0
Undisclosed	14	6.2
How Often Do Patients Bring Up R/S?		
Rarely or Never	28	12.4
Sometimes	135	60.0
Often	39	17.3
Very Often	8	3.6
Undisclosed	15	6.7
Currently Seeing Patients		
Yes	183	81.3
No	28	12.4
Undisclosed	14	6.2
Work Setting^a^		
University Medical Center	75	33.3
Private Hospital/Medical Facility	38	16.9
Public Hospital/Medical Facility	37	16.4
Diagnostic Laboratory – Commercial, Non‐academic	13	5.8
Physician's Private Practice	11	4.9
Not‐for‐profit organization	6	2.7
Diagnostic Laboratory – Non‐commercial, Academic	4	1.8
Telegenetics Company	4	1.8
Pharmaceutical company	3	1.3
University Non‐Medical Center	3	1.3
Government Org./Agency	2	0.9
Diagnostic Laboratory – Commercial, Academic	2	0.9
Health Maintenance Organization	2	0.9
Internet/Website company	1	0.4
Private Practice‐self‐employed	1	0.4
Professional Organization	1	0.4
Other	7	3.1
Undisclosed	15	6.7
Religious/Spiritual Affiliation^a^		
Agnostic	55	24.4
Atheist	32	14.2
Roman Catholic	26	11.6
Jewish	22	9.8
Christian (Disciples of Christ)	18	8.0
United Methodist	12	5.3
None	12	5.3
Presbyterian	11	4.9
Baptist	9	4.0
Unitarian	9	4.0
Lutheran	7	3.1
Episcopalian	5	2.2
Buddhist	3	1.3
Friends (Quaker)	2	0.9
Hindu	2	0.9
Mormon	2	0.9
Greek Orthodox	1	0.4
Muslim	1	0.4
Other	26	11.6
Undisclosed	14	6.2
Currently Practicing Religion		
Yes	121	53.8
No	90	40.0
Undisclosed	14	6.2
Work Specialty^a^		
Prenatal	78	34.7
Cancer	73	32.4
Pediatrics	41	18.2
General Genetics	32	14.2
Preconception/Reproductive Screening	30	13.3
Laboratory	18	8.0
Cardiology	15	6.7
Research	13	5.8
Education: Public or Professional	11	4.9
Neurogenetics	11	4.9
Metabolic Disease/ Lysosomal Storage	10	4.4
Infertility, ART/IVF	9	4.0
Newborn Screening	9	4.0
Genomic Medicine	8	3.6
PGD	7	3.1
Specialty Disease	7	3.1
Administration	5	2.2
Hematology	3	1.3
Pharmacogenetics	3	1.3
Public Health	2	0.9
Psychiatric	1	0.4
Other	11	4.9

Variable	*n*	*M*	*SD*	*Mdn*	Range
Age	89	35.30	11.37	32	23–72
Years of Experience	209	7.61	8.66	4	1–37
Patients per Week	206	11.45	6.24	10	0.5–35
Years Since Last Patient	27	4.52	5.13	2	1–22

*Note*: Percentages may not total 100 due to rounding.

^a^ Participants could endorse all items that applied.

**TABLE 3:** Mean and standard deviation and ANOVA differences by response type and scenario

Variable	Deferring scenario				Attributing scenario				*F*	*p*	$\eta_{p}^{2}$
	*M*	*SD*	*Mdn*	Range	*M*	*SD*	*Mdn*	Range			
Response Type											
Content Response	24.99	33.13	0	0–100	10.53	27.06	0	0–100	24.05	< 0.001	0.11
Open Question	21.88	34.81	0	0–100	30.16	40.83	0	0–100	3.85	0.05	0.02
Closed Question	15.47	27.30	0	0–100	4.99	17.28	0	0–100	19.94	< 0.001	0.09
Influence	14.71	23.02	0	0–100	36.09	36.51	33.3	0–100	59.75	< 0.001	0.23
Information Giving	12.43	23.01	0	0–100	5.25	12.62	0	0–66.7	17.65	< 0.001	0.08
Self‐Disclosure	4.98	15.05	0	0–100	4.15	15.76	0	0–100	0.39	0.53	0.00
Self‐Involving	2.98	10.37	0	0–50	4.11	13.04	0	0–100	0.82	0.37	0.00
Advice	2.00	7.25	0	0–50	1.62	7.30	0	0–50	0.09	0.77	0.00
Affective Response	0.32	3.66	0	0–50	2.67	11.81	0	0–100	7.65	0.006	0.04
Total Thought Units	2.71	1.64	2	1–8	2.66	1.99	2	1–12	0.01	0.91	0.00

The sample varied widely regarding R/S affiliation. The most prevalent responses were agnostic (24.4%) and atheist (14.2%). The remaining participants reported a variety of R/S affiliations. Slightly more than half of the sample (53.8%) reported currently practicing their religion. When asked how often their patients have brought up R/S, the most prevalent response was “sometimes” (60%). When asked about their comfort in discussing R/S with patients, the sample was evenly divided, with 44.8% reporting either *Little or not at all comfortable* or *Somewhat comfortable*, and 48.9% reporting either *Comfortable* or *Very comfortable*.

### 3.2 Genetic counselor response type to prenatal patient's R/S statements

There were 434 responses for the two prenatal scenarios (218 for the Deferring scenario, and 216 for the Attributing scenario). Seven participants did not provide a response to the Deferring scenario, and 9 participants did not provide a response to the Attributing scenario. Each of the nine HSVRS response types occurred in both scenarios (see Table 1 for illustrative examples). Table 3 contains descriptive statistics for each response type and for total thought units separated by scenario. The mean number of total thought units was 2.71 (*SD* = 1.64; Range: 1–8) for the Deferring scenario and 2.66 (*SD* = 1.99, Range: 1–12) for the Attributing scenario. The mean proportionate use of each of the nine response types varied widely within and across scenarios. For the deferring R/S statement, there were greater proportions of content responses which are reflections of the patient's situation, closed questions, and information giving. In contrast, for the attributing R/S statement, there were greater proportions of influencing responses which are attempts to alter the patient's viewpoint or provide encouragement, open‐ended questions, and affect responses which are reflections of patient feelings.

### 3.3 Do response types differ as a function of the content of the patient's R/S statements?

The first MANOVA found a significant multivariate effect for presentation order of the scenarios [Wilks' *λ* = 0.825, *F*(20, 186) = 1.96, *p* = 0.01, $\eta_{p}^{2}$ = 0.18]. Significant univariate effects were found for four dependent variables. When the Deferring scenario was presented first, participants averaged more thought units for both the Deferring scenario (*M* = 2.88 vs. 2.42, *p* = 0.05, $\eta_{p}^{2}$ = 0.02) and the Attributing scenario (*M* = 2.99 vs. 2.21, *p* = 0.006, $\eta_{p}^{2}$ = 0.04) and had a higher percentage of influencing statements in the Attributing scenario (*M* = 40.62 vs. 29.40, *p* = 0.03, $\eta_{p}^{2}$ = 0.02). When the Attributing scenario was presented first, participants had a higher percentage of open‐ended questions in the Attributing scenario (*M* = 38.08 vs. 23.52, *p* = 0.01, $\eta_{p}^{2}$ = 0.03).

The second MANOVA yielded a significant multivariate effect for scenario [Wilks' *λ* = 0.5610, *F*(10, 196) = 12.55, *p* < 0.001, $\eta_{p}^{2}$ = 0.39], but no significant effect for presentation order [Wilks' *λ* = 0.942, *F*(10, 196) = 1.20, *p* = 0.29, $\eta_{p}^{2}$ = 0.06] or the interaction [Wilks' *λ* = 0.909, *F*(10, 196) = 1.97, *p* = 0.04, $\eta_{p}^{2}$ = 0.09]. Follow‐up ANOVAs indicated six statistically significant differences in response types based on scenario (see Table 3 for full results). Compared to the Attributing scenario, response types to the Deferring scenario contained a significantly greater proportion of content response type (*M* = 24.99 vs. 10.53, *p* < 0.001, $\eta_{p}^{2}$ = 0.11); closed questions (*M* = 15.47 vs. 4.99, *p* < 0.001, $\eta_{p}^{2}$ = 0.09); and information giving (*M* = 12.43 vs. 5.25, *p* < 0.001, $\eta_{p}^{2}$ = 0.08). Compared to the Deferring scenario, response types to the Attributing scenario contained a significantly greater proportion of open questions (*M* = 30.16 vs. 21.88, *p* = 0.05, $\eta_{p}^{2}$ = 0.02); influencing response type (*M* = 36.09 vs. 14.71, *p* < 0.001, $\eta_{p}^{2}$ = 0.23); and affective response type (*M* = 2.67 vs. 0.32, *p* = 0.006, $\eta_{p}^{2}$ = 0.04).

#### 3.3.1 Ancillary findings

It was noted that participants were more likely to use R/S language in the Deferring scenario than the Attributing scenario [66.5% vs. 37.5%; *χ* ^*2*^(1, *n* = 210) = 13.10, *p* < 0.001]. Examples of responses containing R/S language in each scenario are as follows: Deferring scenario – “It sounds like your faith is an important part of how you understand what is happening with this pregnancy. Can you tell me more about how God has impacted the way you are coping with this information?”(Participant 2); and “Do you believe that God might change this likely diagnosis before your child is born?”(Participant 10); Attributing scenario: “Yes, it's hard to believe and it must be awful to feel like God is punishing you. Is there something you did that you think deserves punishment?”(Participant 215); and “Neither of us have the ability to understand why painful things like this happen. Genetically, this happens completely by chance, but it obviously doesn't feel random to you in this moment. Have you considered the possibility that God has given you this challenge because he knows that you have the strength to bear it?”(Participant 210).

### 3.4 Are there differences in response types due to counselor comfort level discussing R/S issues?

The MANCOVA indicated that controlling for participant comfort did not affect any of the nine response types [Wilks' λ = 0.955, *F*(10,191) = 0.91, *p* = 0.53, $\eta_{p}^{2}$ = 0.04]. Chi‐square tests indicated no significant difference in the proportion of responses containing R/S language based on level of comfort with discussing R/S issues for either the Deferring scenario [*χ* ^*2*^(3, *n* = 210) = 5.63, *p* = 0.13] or the Attributing scenario [*χ* ^*2*^(3, *n* = 210) = 4.28, *p* = 0.24].

### 3.5 Are there differences in response types as a function of genetic counselor experience?

The MANCOVA analysis also showed that controlling for experience did not affect any of the nine response types [Wilks' λ = 0.904, *F*(10,191), *p* = 0.03, $\eta_{p}^{2}$ = 0.10]. There also was no significant association between having greater than or less than 5 years of experience (based on definition of novice genetic counselors; Zahm et al., 2015) and mentioning R/S language in responses to either scenario [Deferring scenario: *χ* ^*2*^(1, *n* = 207) = 0.75, *p* = 0.39; Attributing scenario: *χ* ^*2*^(3, *n* = 208) = 0.09, *p* = 0.76].

### 3.6 How do genetic counselors explain the reasons for their responses?

Thematic analysis of participants' responses to each scenario revealed nine themes: (1) Exploration, (2) Validation, (3) Normalization, (4) Explaining, (5) Correction, (6) Management, (7) Reassurance, (8) Personalization, and (9) Discomfort. Some counselors provided more than one rationale for their response in each scenario. The response rationales varied with a substantial range in frequencies. Every theme was evident in both scenarios, with the exception of Reassurance, which was only seen in the Attributing scenario. The most frequent rationale for each scenario was Exploration and the least frequent rationale, mentioned by only a few participants, was Discomfort. Table 4 contains a summary of the themes, along with their definitions, frequencies, and illustrative examples of each rationale alongside the responses provided by the participants.

**TABLE 4:** Themes extracted from participants' rationales for their response type in each scenario

Theme and definition	*N*	Example response	Example rationale
Deferring Scenario^a^			
***Exploration*** Clarify and understand/assess the patient's statement, thoughts, and feelings	134	It sounds like you have a strong faith in God. Can you tell me more about what you mean by saying that we will leave things in his hands?	I want to know if this means that they do not need more information because what will happen will happen… or do they mean that they think their baby will be healed. (9)
***Validation*** Acknowledgement and support the patient's statement, beliefs, thoughts, and/or feelings	94	I have heard many patients express similar feelings about a strong faith in God. For some people with this faith, an amniocentesis is not important because they believe that God has control. For others with a strong faith, an amniocentesis is important to know whether the baby has trisomy 18, either so the family can prepare or make pregnancy management decisions. What do you think about when you hear me say this?	*I want to validate the patient's faith*, but also acknowledge that some people with strong faith still undergo an amnio. Even though many people who are religious would not consider termination, I would still want to provide all of the information because I do not know her personal views. (4)
***Correction*** Challenges the patient's misunderstandings or misconceptions.	32	I have a strong faith, too. And I believe that what is God's will will happen and often we do not understand why. There is a chance your baby will be healthy and well. There is also a real chance that your baby will have trisomy 18. Either way I am here to support you along this journey. The amniocentesis will give you more information that may help to prepare you emotionally, and help your doctors to prepare for the delivery. However, the choice is yours – there is no right or wrong answer.	I try not to bring up my faith during genetic counseling sessions unless the patient does. If they bring up their faith, I would like to share this with them and make them aware that we can relate to one another on this spiritual level. *Where I work now, many patients have a strong faith and often think genetic counselors are terrible because we even bring up certain topics*. *I want patients to know that we are here to support them and not to confront them with difficult decisions or options they see as immoral*. (152)
***Management*** Clarify or direct the conversation to medical planning.	25	God may already know what will happen but an amniocentesis will give us a chance to discuss options that will help you and your family plan and prepare for a child that will need additional and special care early on and throughout his/her life.	Acknowledge her belief in omniscience but *review that confirmation may be useful*; that is *development of birthing plan, extended NICU stay, accommodations needed at home for care* (158)
***Explaining*** Prioritize information to help the patient with decision‐making, including making referrals	23	Thank you for sharing with me how you feel. That decision is absolutely fine. While we are concerned about an increased risk to the pregnancy for Trisomy 18, moving forward with additional testing at this time is very much your decision, and we will support you in that.	*This is my attempt to gently reiterate the elevated risk to the pregnancy*, which I felt she understood (i.e., is not in denial), while also supporting her decision. (125)
***Personalization*** Description of participants' own R/S affiliation (or lack thereof) and/or something personal about themselves to justify what they did/did not say to the patient	10	I believe the same thing; that God is able to do anything. And yes, only God knows what will happen today, tomorrow, and next week. Putting all things into his hands can bring unexplainable peace. And sometimes, God puts people in our lives to help us make hard decisions, like this one. I respect whatever decision you make, and I will believe for a miracle with you. But I have learned that sometimes a miracle from God can look different than what we are hoping that miracle to be.	*My belief in God is the anchor of my life; therefore, if someone openly talks about their faith, I will talk God and religion with them*. I would not say these things to someone who does not bring up faith. For this scenario, I would affirm their beliefs, but try to challenge them gently on other thoughts…as there are many things with our faith and God that are not black and white. And I do believe that God performs miracles all the time, they may just not look like what we want them to look like (example: a pregnancy with trisomy 18 – the patient may hope the miracle is their baby is healed … but in reality, the miracle is the baby making it to delivery and the family getting to spend hours/a day before the passing). (89)
***Normalization*** Communicate that the patient's experiences and/or statements are not uncommon	9	It sounds like your faith is very important to you and many families that I work with express the same thoughts that you just did.	I am trying to normalize that the decision to not pursue an amnio is okay. (2)
***Discomfort*** Communicates that participants were not comfortable addressing R/S or that it is out of their scope	8	Because I am required to give you all options, my job is to let you know that termination is an option in this state up to 21 weeks and 6 days. I have no opinion about the right path for you but if you would like more information about termination I can give you that information.	When one of my patients starts talking about God's will I take that to mean they are planning to continue the pregnancy no matter what. I address termination at that time while they are talking about God so I can move past that issue. If they are continuing then I can be free to validate their feelings about God and if they are planning to terminate then I can validate their having to consider a path that they never thought they would be on. *I do not feel comfortable relating on a religious level with patients*. (217)
Attributing Scenario			
***Exploration*** Clarify and understand/assess the patient's statement, thoughts, and feelings	122	Why would God punish you?	I think it is important for the patient to talk about this more, so I think an open‐ended question to gather more information would be effective. (5)
***Validation*** Acknowledgement and support the patient's statement, beliefs, thoughts, and/or feelings	53	I am so sorry that this news is so difficult. It is common for people to try to find an explanation for how something like this could happen to them. Can you tell me more about your faith and why you think God is punishing you?	Again, *I want to validate the patient's concern and allow her to feel comfortable* in talking with me about her faith. Her religion may play an important role in her decision‐making, so it is important that I understand her experience with religion. (4)
***Correction*** Challenges the patient's misunderstandings or misconceptions	48	It sounds like religion is something that is important to you. Tell me more about why you think God is punishing you.	*Ultimately, I would want to address the fact that T18 is most often sporadic meaning Ariana did not do anything to cause this*. However, I would not lead with this information. Instead, I would want to acknowledge what Ariana has just said and ask her to elaborate more on this statement. If I do not explore this with her first and simply tell her this was a random event and not something she caused, she will likely not believe me and disregard my explanation. (6)
***Reassurance*** ^**c**^ Comfort the patient by stating it was not her fault or God's doing and/or to alleviate the patient's guilt	41	Ariana, this is not because of anything you did or did not do. There's always a chance with any pregnancy of a genetic change that leads to birth defects. It's unfair and senseless, but there's nothing you did wrong. There are unfortunately a lot of women who go through similar situations, but it can be very helpful to meet with them for support. If you are interested, I'm happy to find a group or an individual to talk to.	*I wanted to help assure her that this is not her fault*, but I wanted to at the same time steer a bit clear of getting too much into the science and biology of trisomies in case she thought I was dismissing the role God had in her situation. She also seems quite distressed (without having any body language to go off of) so I wanted to offer some support in the form of a support group or one‐on‐one meeting. (52)
***Normalization*** Communicate that the patient's experiences and/or statements are not uncommon	24	It is normal to wonder why this is happening or to feel like we are to blame. It is important to remember that Trisomy 18 occurs by chance, it is not something that anyone chooses.	*It is sometimes hard to know the underlying reason for these comments but often it seems as though the person is feeling guilty or to blame*. *So that is why I would start by normalizing her feeling of questioning*. It provides the genetic counselor a good opportunity to discuss those feelings of guilt or blame. (28)
***Explaining*** Prioritize information to help the patient with decision‐making, including making referrals.	23	Unfortunately, we do not have an explanation as to why this is happening specifically to you, and I know that this is a hard situation that you are in. Our current understanding is that the risk of this happening increases as women get older, but we cannot tell why it happens to one woman rather than another.	I am not a therapist and cannot help her to unpack why she feels that God is punishing her. I can only give her scientific facts to help her understand her situation. (7)
***Personalization*** Description of participants' own R/S affiliation (or lack thereof) and/or something personal about themselves to justify what they did/did not say to the patient	14	If this result is true and the baby does have Trisomy 18, I can assure you that this is not a result of anything that you did or did not do. When I hear responses like this, I find it is oftentimes a reaction of trying to make sense of the occurrence of a very difficult, unexpected, and random event. Unfortunately, parents have a tendency to blame themselves in these situations, which can mean drawing lines between unrelated events.	Clearly, she sees this event as a negative occurrence and punishment. Whether you want to call it karma, or God, or some other force, clearly she feels like there must be a reason for this event to have happened to her. In her case, the only explanation is that this is a punishment for something that happened in her past. You do not necessarily need to address God to let them know that this is not their fault. *I find that telling people what God did or did not intend usually does not go over well, especially coming from someone who is not religious*. (25)
***Discomfort*** Communicates that participants were not comfortable addressing R/S or that it is out of their scope	5	I know that this can be a lot of information to take in all at once. It sounds like you may be feeling a little overwhelmed? Trisomy 18 is something that occurs purely by chance in the developing embryo. Do you think it would help to speak with your [religious leader] or other members of your [religious institution] about all that's been going on? I feel confident that they would assure you that God is not punishing you for something you did.	*As a non‐religious person, this was SUPER hard to come up with a response to! I wanted to say that trisomy 18 is just a chance occurrence – even if to her it is “by God's hand.” I do not feel that it's within my abilities or scope to speak about any religion's ideas about sin, punishment, or why bad things happen*, so that's why I recommended she consider speaking with people more equip to answer her questions. I know it may not be true 100% of the time, but from what I know of religion, I do not feel like the religious institution's stance would be that she did something bad and now is going to have a baby with trisomy 18. (98)
***Management*** Clarify or direct the conversation to medical planning.	4	There is nothing you did to cause this and nothing you could have done to prevent this. These types of conditions truly happen by chance. Now that we have this information we can focus on what is best for you and your baby.	While I would not directly “correct” or “challenge” her statement, because I cannot begin to determine her interpretation of her religion, I want to make sure she hears that this is not her fault. *I would then try and focus on the positive of the situation, which is having this knowledge now so we can plan and prepare appropriately*. (146)

*Note*: Participant numbers presented in parentheses after each quote. Rationales were coded into multiple themes when appropriate; thus, column totals will not be equal to total number of participants. When rationales contained multiple themes, the italicized portion of the rationale is what was representative of the theme.

^a^ Total *N* = 212 participants.

^b^ Total *N* = 210 participants.

^c^ Seen only in Attributing scenario.

## 4 DISCUSSION

This study examined genetic counselors' response types and reasons for their responses to two prenatal patient‐initiated R/S statements. Two hundred twenty‐five genetic counselors responded to two hypothetical scenarios involving a deferring (leave it in God's hands) and attributing (God may be punishing me) R/S statement. Although prior research has investigated patients' R/S beliefs and their effects on genetic counseling processes and outcomes, this is the first investigation of how genetic counselors respond to patient deferring and attributing R/S statements. As the field strives for more culturally inclusive practices, this study provides an initial step in exploring genetic counselors' response types when R/S beliefs arise in sessions.

### 4.1 Genetic counselors' response types to patient's R/S statements

Participants differed in their use of the nine helping skills response types for each patient statement. Content response types, which were prevalent in replies to the deferring R/S statement, encourage patient elaboration/exploration and are considered a form of empathy (Danish et al., 1980; McCarthy Veach et al., 2018). Common content response types included reflections that the patient's faith is a large part of her decision. Perhaps genetic counselors are less inclined to explore a patient's R/S statement that directly contradicts science, as was the case for the attributing R/S statement.

The genetic counselors in the present study were more inclined to use an influencing response type when the patient expressed self‐blame or guilt in the attributing R/S statement. Influencing response types in which counselors attempt to alter patient perspectives are more directive in nature (Danish et al., 1980; McCarthy Veach et al., 2018), which is consistent with the prevalence of influencing responses that stated that trisomy 18 was not the patient's fault; nothing she had done could have caused this. Similarly, Ledford et al. (2015) found physicians' responses differed as a function of the content of patients' R/S statements with physicians giving more control messages, which are similar to influencing response, and less supportive talk messages, which are similar to affective and content response types. In the present study, it appears some genetic counselors similarly wished to exert control and perhaps address the patient's uncertainty (“I don't know why this is happening to me”) by stating an opinion grounded in biomedical expertise about the cause of trisomy 18. Another possibility is they were uncomfortable with R/S causal messages that contradict evidence‐based science and felt obligated to correct the misperception.

In the present study, self‐reported comfort discussing R/S issues varied, although there were no significant differences in the response types given in each scenario or use of R/S language due to the genetic counselors' comfort ratings. These results differ from Reis et al. (2007) who found counselor discomfort was a perceived barrier to conducting spiritual assessments with genetic counseling patients. In the present study however, participants were directed to respond to patient's R/S statements, and it appears that they were able to “set aside” their own comfort/discomfort to do so.

The genetic counselors' use of R/S language differed significantly between the two scenarios. Twice as many genetic counselors used R/S language for the patient's deferring R/S statement than for the attributing statement. One possible explanation is the higher prevalence of content responses to the deferring R/S statement. As content responses essentially reflect the patient's statements, they are more likely to mirror the patient's R/S language. Another possible explanation is the counselors were more comfortable using R/S language in response to the deferring statement because they perceived her statement that “God makes everything possible” to be a positive coping strategy, as opposed to the attributing statement “God is punishing me” which they may have viewed as a negative strategy and/or as scientifically incorrect.

There were no significant differences in the type of genetic counselor responses or in the mention of R/S as a function of their genetic counseling experience. These results are consistent with Reis et al.'s (2007) findings that experience did not influence genetic counselors' spiritual assessment practices in sessions. Further studies should explore possible associations between genetic counselor experience and their response types to patients' R/S statements.

It is worth noting that this study examined genetic counselor response type via the HSVRS. A thematic analysis of responses was not performed given the brief nature of genetic counselor responses that precluded this deeper analysis. While the purpose of this study was not to examine response quality or motivations, it is interesting to consider how genetic counselor's responses to R/S statements may be intentionally brief in nature. This could reflect a number of factors: the lack of comfort that influenced a more truncated discussion; the conflict between the teaching and counseling model of genetic counseling practice; and/or the lack of time that logistically prevents genetic counselors from more deeply addressing psychosocial topics. Another possible explanation is that participants were asked to provide a single response; thus, it is unknown whether they would have further discussed R/S in an actual session. There are potentially endless hypotheses, and yet, it may be that the cursory responses collected in this study accurately reflect the various ways in which genetic counselors navigate R/S when it arises in sessions.

Overall, the wide variation in response types found in this study indicates that there is no prescribed way to respond to patient's deferring or attributing statements in the context of R/S discussions. Practicing genetic counselors, as well as trainees, may find freedom in their ability to respond in a patient‐centered and tailored way to patient's discussion of their R/S beliefs. Training and education should similarly underscore the variety of ways in which genetic counselors might respond and identify possible advantages and disadvantages of different approaches for different patients. For instance, content and affective statements are “continuing responses” intended to promote elaboration of thoughts, feelings, and experiences of concern to the patient (Danish et al., 1980; McCarthy Veach et al., 2018). Questions, influencing responses, advice, and information statements, are “leading responses,” that are intended to guide the conversation in directions of concern to the genetic counselor. Self‐disclosure and self‐involving statements are “self‐referential” responses intended to respectively share personal information and here‐and‐now reactions of the counselor. Consideration of concrete examples of these different response types to a patient's R/S statement can be used as a helpful starting framework, especially for trainees and novice genetic counselors.

### 4.2 Genetic counselors' explanations of their responses

Participants provided a variety of reasons for their responses with exploration being most prevalent in both scenarios. Exploration is consistent with the use of content and affective responses, as well as with open‐ and closed‐ended questions (Danish et al., 1980; McCarthy Veach et al., 2018), which were highly prevalent in the participants' response types. This reason is also congruent with studies showing the significant role R/S plays in terms of coping, risk perception, and decision‐making by patients and families (Grossoehme et al., 2010; Quillin et al., 2006; Seth et al., 2011). Exploring patients' R/S statements allows the counselor to assess and better understand the patient's underlying thoughts, feelings, and beliefs which, in turn, may positively affect genetic counseling processes and outcomes. Studies have found that medical patients who had R/S discussion rated overall experience and satisfaction higher, and some patients want their genetic counselors to explore their R/S beliefs and values in the context of their healthcare (Sagaser et al., 2016; Williams et al., 2011). These findings reflect the tenets and goals of the Reciprocal‐Engagement Model (REM) of genetic counseling practice (McCarthy Veach et al., 2007) that stress the importance of understanding patients' emotions and their cultural and familial contexts as well as the importance of assessing and addressing patients' feelings and beliefs (Redlinger‐Grosse et al., 2017).

Explaining and correction were also relatively common reasons given for participants' response types to both scenarios and are consistent with the prevalence of counselor information giving and influencing responses by participants (combined average of 27.14% for Deferring scenario and 41.34% for Attributing scenario). Provision and interpretation of biomedical information comprise a key tenet of the REM (McCarthy Veach et al., 2007). Ernecoff et al. (2015) similarly found healthcare professionals typically respond to patients' R/S statements by talking about the medical plan or goals of care. These reasons are congruent with previous studies showing that a large proportion of communication in genetic counseling focuses on biomedical information and education rather than psychosocial issues (Meiser et al., 2008; Paul et al., 2015). There exists, however, a difference in explaining versus correction. While genetic counselors may feel a high sense of responsibility to correct misinformation, one could argue that correction may come across as lacking empathy and/or cultural sensitivity. Further research should examine patients' preferences for different genetic counselor responses to R/S statements and their reactions to statements. Additionally, the timing of responses associated with these reasons may be critical. Statements intended to provide and/or interpret biomedical explanations may have limited impact as an initial response (e.g., Hornsey & Fielding, 2017). Perhaps genetic counselors should first explore patients' underlying beliefs, feelings, and attitude roots before moving on to explaining, correction, or management. Research is needed to investigate this hypothesis.

There were two apparent thematic differences in reasons given based on scenario. Validation appeared to be more common for the Deferring scenario, which makes sense as the deferring patient's R/S statement implied a degree of acceptance, that is, the patient seemed at peace with leaving things in God's hands. Reassurance emerged as a reason only for the Attributing scenario. As the patient's R/S statement in this scenario had undertones of self‐blame and guilt, it is not surprising that many genetic counselors wished to provide reassurance. Reassurance aligns with the most prevalent type of genetic counselor response type for this scenario – influencing responses. While beyond the scope of this study, it would have been interesting for genetic counselors to reflect on their differing rationales based on scenarios to explore the intentionality of their choices. Given the variation in response types and rationales in this study, one may hypothesize that responses are based on more personalized frameworks, rather than informed or empirically based choices. Training that increases genetic counselor self‐awareness and cultural biases while also providing contexts for genetic counselors to understand the importance and function of R/S in healthcare would provide an important framework to draw from in their psychosocial counseling.

Finally, although participants' ratings of their comfort with discussion of R/S were not a significant factor in the current study, it is interesting to highlight that some genetic counselors identified their discomfort as a reason for their response in one or both scenarios. They variously noted their discomfort originated from a lack of an R/S affiliation, lack of training regarding how to address patient's R/S, belief that R/S discussions were outside the scope of genetic counseling practice, and their disagreement with the patient's R/S sentiments. Reis et al. (2007) also reported these reasons as barriers to R/S assessment. Given cultural competency is one of the cornerstones of genetic counseling, R/S discussions arguably are within the scope of practice (Wang, 1994; Warren, 2011a; Warren, 2011b; Weil, 2001). Almost 40% of the present sample (38.6%) identified themselves as agnostic or atheist, which is consistent with a recent study of genetic counseling students (37.9%, Stoddard et al., 2020). Further studies could investigate genetic counselors' reactions and responses when their R/S views are opposed to those of their patients.

Education regarding the role of religion and spirituality in patient care and ways to address it in sessions may increase genetic counselor comfort with such discussions in their practice (Murray et al., 2020) and reinforce that R/S discussions are within the scope of genetic counseling practice. Education also reinforces the importance of culturally inclusive care in healthcare. Practice opportunities such as role plays for students to specifically engage in R/S discussions in a deliberative may assist in increasing comfort (Murray et al., 2020).

It should also be considered that participants' reported discomfort could have stemmed from a countertransferential reaction to the patient statement(s) such as feeling they are not qualified to discuss R/S beliefs, concern they might unduly influence the patient, or feeling unable to connect empathically with them. The importance of self‐reflection in promoting awareness and management of countertransferential reactions that may arise in discussions of R/S beliefs is warranted. Self‐reflection has been shown to be a valuable tool in genetic counseling practice (e.g., Runyon et al., 2010), and as such, this practice can begin during training. Training programs can provide opportunities for introspection to enhance awareness of their own motivations, understand reactions generated by interactions with patients, and enhance the way they respond to R/S statements (Christensen et al., 2018; Ernecoff et al., 2015). Continued reflection opportunities through peer support/consultation may be one effective vehicle to promote self‐reflection for practicing genetic counselors (e.g., Lewis et al., 2016; Zahm et al., 2007).

Again, overall, the participant's varied explanations of their responses even within these two scenarios indicate the unique intentions and perspectives of genetic counselors on R/S discussions. Genetic counselors see their role and responsibility in responding to R/S statements quite differently – from empathic, to corrective, to discomfort. Regardless, the intentionality of how and why they address patient's R/S statements remains critical.

### 4.3 Study limitations

There are several study limitations that may restrict generalizability of the findings. It is unknown whether survey respondents differ from non‐respondents. Less than half of the sample responded to a demographic item asking their age. While it is unclear why this occurred, it likely does not impact the interpretation of our findings. The sample does not reflect the 2018 PSS in terms of practice specialty, with prenatal being higher than cancer in this study. This difference is likely due to the prenatal focus of the scenarios attracting a greater number of prenatal genetic counselors. This prenatal focus also means the findings might differ for other practice specialties. Furthermore, given the hypothetical nature of the scenarios, the responses might not reflect actual genetic counseling interactions in which genetic counselors lack the opportunity to spend as much time as they wish to consider and revise their responses. Written responses also lack non‐verbal nuances that are powerful aspects of any message. Additional limitations concern the HSVRS which categorizes only the type of helping response; it does not assess response quality or the motivations behind the responses. Future research is needed that would allow for analysis of quality and a deeper consideration of the participants' response motivations. With regard to the HSVRS, this system requires specific training and there are few who are certified in its use, which makes assessment of its reliability challenging for individual studies. Our use of a criterion‐trained rater provides good evidence of the system's reliability, but the use of multiple such trained individuals to establish the reliability of the system in genetic counseling research would be a valuable contribution to the literature.

This study also does not address whether the responses provided by participants are specific to R/S patient statements. For example, participants may make similar responses if the patient had framed her statement in terms of luck. Future studies could examine how genetic counselor response might vary according to R/S vs. non‐R/S statements. Participants' rationales for their responses also only reflect conscious attributions for their behavior. Additional factors, such as implicit bias, social desirability, and cognitive dissonance, may also be playing a role, as considerable research from social psychology indicates situational factors strongly influence behavior. Future studies would benefit from manipulating such factors to attempt to isolate unconscious factors affecting behavior. Additionally, single responses to one patient statement do not capture the nature and cumulative effect of a more extended discussion. Finally, we acknowledge the potential for observer bias in analyses, including the HSVRS system, coding of responses and religious/not religious, and thematic analysis given the researchers were not blinded in the coding process.

### 4.4 Training and practice implications

As discussed above, the present findings suggest several training and practice implications. For trainees and practicing genetic counselors, the wide variety of response types obtained in this study illustrate different ways genetic counselors respond to the same patient's R/S statement. Awareness of this variability can liberate genetic counselors from the notion that there is a “correct” type of response to patient's R/S statements. The importance of deliberative and self‐reflective practice in responding to R/S statements is another important takeaway from the study findings. This intentionality can be fostered within training programs as discussion of cultural factors such as R/S that inform patient's thoughts, beliefs, and reactions to genetic information. Role plays and peer consultation are suggested opportunities to foster comfort, versatility, and deliberateness in addressing R/S statements. Finally, training can continue to stress the importance of fostering relationships with hospital chaplaincy, as well as other ancillary healthcare members, as a place to continue to build cultural humility in working with individuals and families where R/S plays a major role in their experience of genetic counseling (Puchalski et al., 2014).

### 4.5 Research recommendations

This study is an initial step toward understanding how genetic counselors respond to patient's R/S statements in prenatal genetic counseling sessions. As discussed throughout, the present findings suggest several avenues for future research. While this study found that counselors respond to patient's R/S statements in a variety of ways, the effects of the type of responses are unknown. Additional research should assess the effectiveness of different types of responses and the reactions these responses elicit in patients. Qualitative investigations should be done to examine in greater depth factors that influence genetic counselors' reactions and responses to patient's R/S statements. In the present study, experience level approached statistical significance, and therefore, more studies of the impact of experience on genetic counselor responses are warranted. Additional factors that merit further exploration of their effects on counselor responses include counselor R/S affiliation, practice specialty, patient cultural characteristics, and the nature of the R/S statements (e.g., deferring, attributing, conflicting).

## 5 CONCLUSION

Research shows R/S influence risk perception, decision‐making, and coping by patients and families. This exploratory study explored the type of genetic counselor responses to a prenatal patient's deferring and attributing R/S statements in two hypothetical genetic counseling scenarios and the reasons for their responses. Results indicate a wide range of genetic counselors' response types that differed based on the content of the R/S statements, as well as varied reasons given for the responses. Responses did not differ significantly as a function of genetic counselor comfort with R/S discussions or years of experience. The findings reflect genetic counselor stylistic differences in how and why they respond to patients. Given patients also differ, genetic counselors continue tailor their counseling in a person‐centered manner and emphasize the centrality of the patient‐counselor relationship (McCarthy Veach et al., 2007). Genetic counseling cannot be manualized. We need to find ways to address and incorporate our patients' beliefs into our counseling that are respectful and supportive. As genetic counselors continue in their efforts to address the diverse needs of our patients in a culturally inclusive manner, continued examination through training and research on responses to R/S will ultimately enhance genetic counseling processes and outcomes.

## AUTHOR CONTRIBUTIONS

**Krista Redlinger‐Grosse:** Conceptualization; data curation; formal analysis; investigation; methodology; writing – original draft. **Alina Sitaula:** Conceptualization; data curation; formal analysis; investigation; methodology; writing – original draft; writing – review and editing. **Patricia McCarthy Veach:** Conceptualization; methodology; supervision; writing – original draft; writing – review and editing. **Ian M. MacFarlane:** Conceptualization; formal analysis; methodology; writing – original draft; writing – review and editing. **Whiwon Lee:** Conceptualization; methodology; writing – review and editing.

All of the authors gave final approval of this version to be published and agree to be accountable for all aspects of the work in ensuring that questions related to the accuracy or integrity of any part of the work are appropriately investigated and resolved. All authors reviewed and approved the revised and resubmitted manuscript.

## Conflict of interest

Alina Sitaula, Patricia McCarthy Veach, Ian M. MacFarlane, Whiwon Lee, and Krista Redlinger‐Grosse declare they have no conflict of interest.

## Human studies and informed consent

All procedures followed were in accordance with the ethical standards of the responsible committee on human experimentation (institutional and national) and with the Helsinki Declaration of 1975, as revised in 2000 (5). Informed consent was obtained from all participants for being included in the study.

## Animal studies

No animal studies were carried out by the authors for this article.

## Supporting information


Appendix S1

## Data Availability

Data for this study are available upon request from the corresponding author. All data obtained during this study that are relevant to the research questions are reported in the published article, tables, and supplemental material.
