# Supplementary material for: Genetic counselors' response types to prenatal patient deferring or attributing religious/spiritual statements: An exploratory study of US genetic counselors
Source: J Genet Couns. 2022 Sep 21;32(1):197–212. doi: 10.1002/jgc4.1634 (PMC10087964; doi:10.1002/jgc4.1634)
Supplement: Supplementary file 1 — Appendix S1 [file JGC4-32-197-s001.docx]

# **Supplemental Appendix: Survey**

## **Genetic Counseling Scenarios and Dialogue**

The following sections contain two hypothetical genetic counseling scenarios and dialogues. Each of the scenarios is the same with a *slightly* different dialogue.

**Please imagine that you are the genetic counselor in each of these hypothetical scenarios and dialogues.**

**Scenario 1:**

Ariana is a 35-year-old woman referred for genetic counseling at 18 weeks gestation. This is her first pregnancy. Her obstetrician ordered a Non-Invasive Prenatal Test (NIPT) because of advanced maternal age, and the results came back positive for trisomy 18. Prior to her meeting with you today, she had a level 2 ultrasound, and clenched fists and choroid plexus cysts were found.

You are meeting with her after her ultrasound and believe you have established rapport and a good working relationship with Ariana. You have reviewed with her the NIPT result, the ultrasound findings, and the clinical features of trisomy 18. You have gone over the difference between screening and diagnostic testing and have talked with Ariana about amniocentesis and its benefits and limitations. She indicates that she has no additional questions about the information you presented, and you assess her understanding of the information to be quite good. You are now discussing Ariana’s decision about whether or not to pursue amniocentesis, and she says:

*Ariana:* Is there any chance that this blood test result is wrong?

*You [Genetic Counselor]:* Because NIPT is only a screening tool, there’s always a small chance of false positives. However, based on the ultrasound results, the chance that this result is wrong is very low.

*Ariana:* (Brief silence) … Okay.

*You [Genetic Counselor]:* If you would prefer a definite answer on whether the pregnancy has trisomy 18, amniocentesis is the only option. (Pause)… What are your thoughts at this point?

*Ariana:* **I don’t know. (Pause)… God makes everything possible. Only God knows what can happen. We leave things in his hands.**

1. As the genetic counselor, please write what you will say next to Ariana, **as if you are actually talking to her.**

You [Genetic Counselor] say:

1. Please provide an explanation of your response.

**Scenario 2:**

Ariana is a 35-year-old woman referred for genetic counseling at 18 weeks gestation. This is her first pregnancy. Her obstetrician ordered a Non-Invasive Prenatal Test (NIPT) because of advanced maternal age, and the results came back positive for trisomy 18. Prior to her meeting with you today, she had a level 2 ultrasound, and clenched fists and choroid plexus cysts were found.

You are meeting with her after her ultrasound and believe you have established rapport and a good working relationship with Ariana. You have reviewed with her the NIPT result, the ultrasound findings, and the clinical features of trisomy 18. You have gone over the difference between screening and diagnostic testing and have talked with Ariana about amniocentesis and its benefits and limitations. She indicates that she has no additional questions about the information you presented, and you assess her understanding of the information to be quite good. You are now discussing Ariana’s decision about whether or not to pursue amniocentesis, and she says:

*Ariana:* Is there any chance that this blood test result is wrong?

*You [Genetic Counselor]:* Because NIPT is only a screening tool, there’s always a small chance of false positives. However, based on the ultrasound results, the chance that this result is wrong is very low.

*Ariana:* (Brief silence) … Okay.

*You [Genetic Counselor]:* If you would prefer a definite answer on whether the pregnancy has trisomy 18, amniocentesis is the only option. (Pause)… What are your thoughts at this point?

*Ariana:* **I don’t understand why this is happening to me. (Pause)… I feel like God is punishing me for something I did.**

1. As the genetic counselor, please write what you will say next to Ariana, **as if you are actually talking to her.**

You [Genetic Counselor] say:

1. Please provide an explanation of your response.

**Participant Demographics**

1. What is your gender?
   - Female
   - Male
   - Non-binary
   - Transgender Male
   - Transgender Female
   - Other
   - Prefer not to disclose
2. What is your current age? ______
3. What is your ethnic background?
   - African

- American Indian or Alaskan Native
- Asian
- Black or African American
- Bi-racial (Please specify): ___________
- Caucasian or White
- Hispanic/Chicano/Latina(o)
- Native Hawaiian or Other Pacific Islander
- Other (Please specify): _________

1. With which religious or spiritual affiliation do you most identify? (check all that apply)
   - Agnostic

- Atheist
- Baptist
- Buddhist
- Christian (Disciples of Christ)
- Episcopalian
- Friends (Quaker)
- Greek Orthodox
- Hindu
- Islamic
- Jehovah’s Witness
- Jewish
- Lutheran
- Mormon (Church of the Jesus Christ of Latter Day Saints)
- Presbyterian
- Roman Catholic
- Scientologist
- Taoist
- Unitarian
- United Methodist
- Other (Please specify): _________
- None

1. Are you currently practicing your religion? (Automatically skipped if selected none or atheist in previous question)

- Yes
- No

1. Do you currently see patients clinically?
   - Yes
   - No
2. (Automatically skipped if selected Yes to previous question): How many years has it been since you last saw patients in a clinical setting? (If less than 1, please put “1”.) ________ year
3. On average, how many genetic counseling patients do/did you see per week? ________
4. On average, how many hours per week do you work? _________ hours
5. How many years have you been working as a genetic counselor? (If less than 1 year, please put “1”.) _________ year
6. Are you a certified genetic counselor or board eligible genetic counselor?
   - Yes
   - No
7. What is your current primary specialty area(s)? (Please check all that apply.)
   - Administration
   - Cancer
   - Cardiology
   - Education: Public or Professional
   - General Genetics
   - Genomic medicine
   - Genomic Profiling/ Personal Genomics
   - Hematology
   - Infertility, ART/IVF
   - Laboratory
   - Metabolic Disease (including Lysosomal Storage)
   - Neurogenetics
   - Newborn Screening
   - Pediatrics
   - Pharmacogenetics
   - Preconception/ Reproductive Screening
   - Prenatal
   - PGD
   - Psychiatric
   - Public Health
   - Specialty disease
   - Research
   - Other (Please specify): ___________
8. Which of the following best describes your primary work setting? (Please check one)

| Bioinformatics Company | Outreach/Satellite/Field Clinic |
| --- | --- |
| Diagnostic Laboratory- Commercial, Academic | Pharmaceutical Company |
| Diagnostic Laboratory – Commercial, Non-academic | Physician’s Private Practice |
| Diagnostic Laboratory – Non-commercial, Academic | Private Hospital/Medical Facility |
| Federal/State/County Office | Private Practice – Self-Employed |
| Government Organization or Agency | Professional Organization |
| Health Advocacy Organization | Public Hospital/Medical Facility |
| Health Maintenance Organization | Research Development/Biotechnology Company |
| Internet/Website Company | Telegenetics Company |
| Marketing/Advertising Company | University Medical Center |
| Not-For-Profit Organization (not otherwise specified) | University Non-Medical Center |
| Other (please specify) |  |

1. Typically, how comfortable are/were you discussing religion and spirituality with your patients?
   - Little or not at all comfortable
   - Somewhat Comfortable
   - Comfortable
   - Very Comfortable
2. Please comment on your rating.
3. In your genetic counseling experience, how often on average do/did patients bring up religion and spirituality?
   - Rarely or Never
   - Sometimes
   - Often
   - Very Often
